# Rasch Analysis of the Arabic Fear-Avoidance Beliefs Questionnaire in Individuals with Low Back Pain

**DOI:** 10.3390/healthcare14182913

**Published:** 2026-09-09

**Authors:** Mishal M. Aldaihan, Abdulrahman M. Alsubiheen, Ali H. Alnahdi

**Affiliations:** Department of Rehabilitation Sciences, College of Applied Medical Sciences, King Saud University, P.O. Box 10219, Riyadh 11433, Saudi Arabia; mishaldaihan@ksu.edu.sa (M.M.A.);

**Keywords:** fear-avoidance beliefs, low back pain, Rasch analysis, measurement properties, patient-reported outcome measures, psychometrics, structural validity

## Abstract

**Background/Objective:** The measurement properties of the Arabic Fear-Avoidance Beliefs Questionnaire (FABQ) have not been examined using the Rasch measurement model. This study evaluated the Physical Activity (FABQ-PA) and Work (FABQ-W) subscales of the Arabic FABQ in individuals with low back pain (LBP). **Methods:** This cross-sectional study included 113 individuals with LBP who completed the Arabic FABQ. The FABQ-PA and FABQ-W were evaluated separately using RUMM2030. Likelihood-ratio tests supported use of the partial credit model for both subscales. Rasch analysis examined overall and individual item fit, person misfit, response-category threshold ordering, local item dependency, differential item functioning (DIF), person separation, unidimensionality, and targeting. DIF was investigated by sex, age, and LBP duration. Unidimensionality was evaluated by comparing person estimates derived from item subsets defined by principal component analysis of residuals. Targeting was examined using person–item threshold distributions. A previously proposed four-category rescoring structure was additionally explored because of disordered thresholds. **Results:** Following removal of participants with substantial person misfit, both FABQ-PA (n = 105) and FABQ-W (n = 106) demonstrated satisfactory overall Rasch model fit, and all individual items showed satisfactory fit. Both subscales supported unidimensionality, with no evidence of local item dependency or DIF by sex, age, or LBP duration. Targeting was generally adequate, although coverage was less optimal at the higher end of FABQ-PA and lower end of FABQ-W. Person separation was limited for FABQ-PA (PSI = 0.60) but good for FABQ-W (PSI = 0.80). All items demonstrated disordered thresholds using the original seven-category response scale. A previously proposed four-category rescoring structure improved, but did not completely resolve, threshold disordering. **Conclusions:** The Arabic FABQ-PA and FABQ-W demonstrated satisfactory final model and item fit and supported unidimensional measurement; however, important limitations were identified. Limited person separation for FABQ-PA and persistent threshold disordering across both subscales indicate that scores should be interpreted cautiously and that further refinement of the FABQ response format is warranted.

## 1. Introduction

Fear-avoidance beliefs are an important psychological factor associated with disability in individuals with low back pain (LBP) [1,2]. According to the fear-avoidance model, individuals who interpret pain as threatening may develop fear of movement, physical activity, or reinjury, leading to avoidance behaviors that can contribute to activity restriction and persistent disability [3,4]. Fear-avoidance beliefs have been associated with LBP-related disability and may have prognostic value, particularly for work-related outcomes [1,2]. Accurate assessment of these beliefs is therefore important in both clinical practice and research.

The Fear-Avoidance Beliefs Questionnaire (FABQ) is one of the most widely used patient-reported measures of fear-avoidance beliefs in individuals with LBP [5]. It comprises two distinct subscales assessing beliefs related to physical activity (FABQ-PA) and work (FABQ-W). The FABQ has been translated and validated across several languages and populations [6,7,8]. Two Arabic versions have also been developed, with evidence supporting their reliability and construct validity [9,10]. The version developed by Alanazi et al. [9] uses Modern Standard Arabic, which is broadly understood across Arabic-speaking populations, rather than a local Arabic dialect as used in the other available Arabic version [10]. More recently, confirmatory factor analysis supported the two-factor structure of the Arabic FABQ, with physical activity and work items representing separate but related constructs [11].

Although factor analysis provides important evidence regarding dimensionality, it does not fully address how individual items and response categories function along the underlying construct. Analysis using the Rasch measurement model provides complementary evidence by examining whether responses satisfy the requirements of a probabilistic measurement model [12,13,14,15]. It allows evaluation of overall and individual item fit, response-category functioning, local item independence, differential item functioning (DIF), reliability, and targeting of item difficulty to respondents’ levels of the measured construct [12,13,14,15]. These properties are particularly relevant to the FABQ because its items use seven response categories ranging from 0 (completely disagree) to 6 (completely agree), including an intermediate unsure category [5].

Previous Rasch analyses have raised concerns regarding the measurement properties of the FABQ. Meroni et al. reported inadequate Rasch model fit for the Italian FABQ and its subscales [16]. Similarly, Aasdahl et al. found that neither FABQ subscale satisfied Rasch model requirements and questioned whether the seven-category response structure provided meaningful distinctions between levels of fear-avoidance beliefs [17]. A subsequent analysis by Franchignoni et al. identified extensive threshold disordering and showed improved measurement properties after modifying the response structure, including collapsing adjacent categories and treating the unsure response as missing [18]. Collectively, these studies suggest that the original FABQ response scale may not function optimally and that its measurement properties may vary across populations.

Despite existing evidence supporting the reliability, construct validity, and two-factor structure of the Arabic FABQ [9,10,11], its item-level measurement properties have not been evaluated using Rasch analysis. Such evaluation is important because measurement properties observed in other language versions cannot necessarily be assumed to apply to the Arabic version, particularly when response-category interpretation may be influenced by linguistic or cultural factors [19]. Therefore, this study aimed to evaluate the measurement properties of the Arabic FABQ-PA and FABQ-W subscales in individuals with LBP using the Rasch measurement model. Specifically, the study examined overall subscale fit, individual item fit, targeting, response category functioning, local item dependency, differential item functioning, reliability, and unidimensionality.

## 2. Materials and Methods

### 2.1. Study Design

This cross-sectional psychometric study employed the Rasch measurement model to evaluate the measurement properties of the Arabic Fear-Avoidance Beliefs Questionnaire (FABQ) in individuals with LBP. Separate Rasch analyses were conducted for the Physical Activity (FABQ-PA) and Work (FABQ-W) subscales because each subscale is intended to measure a distinct dimension of fear-avoidance beliefs. The study was conducted in accordance with the Declaration of Helsinki and received ethical approval from the institutional review boards of King Saud University (E-20-5529) and Security Forces Hospital (22-601-37). All participants provided written informed consent before study participation.

### 2.2. Setting and Participants

Participants were recruited consecutively from the outpatient physical therapy departments of Security Forces Hospital and Alrass General Hospital, two tertiary healthcare institutions located in the central region of Saudi Arabia. Recruitment took place during the participants’ initial physical therapy visit following referral from specialist physicians in primary care, orthopedic, or spine clinics.

Individuals were eligible to participate if they were 18 years of age or older, had a primary complaint of LBP, were referred for physical therapy management, and were able to read and understand Arabic. Participants were excluded if they reported neurological, cardiopulmonary, or systemic conditions that could independently limit physical function and confound the assessment of fear-avoidance beliefs. Individuals with a history of lumbar spine surgery were also excluded. Of the 113 participants included in the present study, 112 (99.1%) were also included in our previously published confirmatory factor analysis of the Arabic FABQ [11], while one additional participant was included in the present analysis. The previous study evaluated the factorial structure of the Arabic FABQ, whereas the present study examined its measurement properties using Rasch measurement theory.

### 2.3. Procedure

Eligible individuals were identified by the treating physical therapists during their initial outpatient visit and were invited to participate in the study. After providing written informed consent, participants completed the Arabic FABQ as part of the baseline assessment before the initiation of physical therapy. Demographic and clinical information, including age, sex, and duration of LBP, was collected at the same visit. LBP duration was categorized as acute (<1 month), subacute (1–3 months), or chronic (>3 months). Work status was also recorded and categorized as employed, housework, unemployed, or retired. Clinical characteristics collected included pain intensity using the 0–10 Numeric Pain Rating Scale (NPRS) [20], disability using the Oswestry Disability Index (ODI) [21,22] (0–100), with higher scores indicating greater pain intensity and disability, respectively.

### 2.4. Outcome Measure

#### Fear-Avoidance Beliefs Questionnaire (FABQ)

Fear-avoidance beliefs were assessed using the Arabic version of the Fear-Avoidance Beliefs Questionnaire (FABQ) developed and validated by Alanazi et al. [9]. This version was selected because it uses Modern Standard Arabic, which is broadly understood across Arabic-speaking populations, rather than a local Arabic dialect as used in the other available Arabic version [10]. The validated Arabic version was administered without modification. The FABQ is a patient-reported questionnaire developed to assess beliefs that physical activity and work may provoke pain or impede recovery in individuals with LBP [5]. The instrument contains 16 statements rated on a seven-point agreement scale from 0 (“completely disagree”) to 6 (“completely agree”) with a score of 3 in the middle representing an “unsure” response. Consistent with the original scoring recommendations, only 11 items contribute to the questionnaire scores, whereas the remaining five items are not included in score calculation [5].

The FABQ generates two distinct subscale scores. The Physical Activity subscale (FABQ-PA) comprises items 2–5 and yields scores ranging from 0 to 24, while the Work subscale (FABQ-W) comprises items 6, 7, 9, 10, 11, 12, and 15 and yields scores ranging from 0 to 42 [5]. Higher scores on either subscale indicate stronger fear-avoidance beliefs within the corresponding domain. All participants completed the FABQ-W items irrespective of current work status, and no participants were excluded from the FABQ-W analysis on the basis of work status. No modification of the FABQ-W items was made according to employment status.

Because the FABQ-PA and FABQ-W are intended to represent distinct dimensions of fear-avoidance beliefs, each subscale was evaluated separately in the Rasch analysis. Previous studies have provided evidence supporting the reliability and construct validity of the Arabic FABQ [9,11], including recent evidence supporting its two-factor structure using factor analysis in individuals with LBP [11]. However, the measurement properties of the Arabic FABQ subscales have not previously been evaluated using the Rasch measurement model.

### 2.5. Statistical Analysis

Rasch analysis was performed separately for the Physical Activity (FABQ-PA) and Work (FABQ-W) subscales using the partial credit model implemented in RUMM2030 software (Version 5.4; RUMM Laboratory Pty Ltd., Perth, Australia) [23,24,25]. Separate analyses were undertaken because the FABQ-PA and FABQ-W are intended to measure distinct dimensions of fear-avoidance beliefs and therefore should independently satisfy the assumptions of the Rasch measurement model. Because Rasch analysis assumes that items analyzed together represent a single underlying construct, a joint analysis of all FABQ items would not be appropriate given the established two-dimensional structure of the instrument. Previous work using confirmatory factor analysis supported the two-factor model of the Arabic FABQ [11]. The appropriate Rasch model parameterization was determined using the likelihood-ratio test [12,26]. A statistically significant likelihood-ratio test indicates that the threshold structure differs across items and supports use of the Partial Credit Model rather than the Rating Scale Model, which assumes a common threshold structure across items.

Overall fit of each subscale to the Rasch model was evaluated using the item–trait interaction chi-square statistic, with a non-significant result indicating invariance of items difficulties across the latent trait and adequate fit to the model [12,26]. Overall model fit was further examined using the mean and standard deviation of the item and person fit residuals. The distributions of item and person fit residuals were also examined, with mean values expected to approximate 0 and standard deviations to approximate 1 under satisfactory model fit [12,26]. No strict numerical acceptable ranges were prespecified for these distributional summary statistics; rather, they were evaluated descriptively in relation to these expected values. Individual item fit was assessed using standardized fit residuals and item-level chi-square statistics. Fit residuals outside ±2.5 were considered indicative of item misfit. The item-level chi-square *p*-values were evaluated against a Bonferroni-adjusted significance level of 0.05 divided by the number of items within each subscale to account for multiple testing [12,26]. Person fit was evaluated using standardized person fit residuals, with values outside ±2.5 considered indicative of substantial person misfit. Participants demonstrating substantial person misfit were identified and removed iteratively, with the Rasch model re-estimated after each stage of removal. Person misfit was examined as part of the prespecified Rasch analytical procedure to identify response patterns inconsistent with model expectations rather than solely as a means of achieving satisfactory overall model fit [12,26].

The performance of the response categories was evaluated by inspecting category probability curves and threshold ordering for each item. Ordered thresholds indicate that respondents can consistently distinguish between adjacent response categories, whereas disordered thresholds suggest that the response categories do not function as intended [12,14,27]. Local item independence was examined using residual correlations between item pairs. Residual correlations exceeding 0.20 were considered indicative of potential local item dependency after accounting for the primary latent construct via the Rasch model [13,28]. Reliability was assessed using the Person Separation Index (PSI), which reflects the ability of the subscale to distinguish between individuals with different levels of the underlying construct [12,25,29]. Higher PSI values indicate greater measurement precision and improved discrimination between levels of fear-avoidance beliefs.

Unidimensionality was evaluated using Smith’s approach based on principal component analysis of the residuals [12,30]. Items loading positively and negatively on the first residual component were used to generate two independent sets of person estimates, which were subsequently compared using *t*-tests. A subscale was considered unidimensional when fewer than 5% of these comparisons were statistically significant or when the 95% binomial confidence interval for the proportion of significant tests included 5% [12,30]. The 95% binomial confidence interval for the proportion of significant *t*-tests was calculated using the Wilson score method [31].

Differential item functioning (DIF) was examined according to sex (male/female), age (≤28/>28 years), and LBP duration (acute/subacute versus chronic). Age was dichotomized at the sample median (28 years) to provide approximately balanced age groups. Acute and subacute LBP were combined because of the relatively small numbers of participants in these categories and to compare participants with LBP duration ≤3 months with those with chronic LBP (>3 months). Uniform and non-uniform DIF were examined using two-way analysis of variance of standardized residuals across three class intervals and each grouping variable [12,15]. A significant main effect of the grouping variable was considered indicative of uniform DIF, whereas a significant interaction between the grouping variable and class interval was considered indicative of non-uniform DIF. The level of significance for the DIF analyses was adjusted for multiple testing using the Bonferroni correction. For each grouping variable, the correction was applied across the three analysis of variance effects (class interval, grouping variable, and class interval × grouping variable interaction) for each item, resulting in 12 comparisons for FABQ-PA (4 items × 3 effects) and 21 comparisons for FABQ-W (7 items × 3 effects). Accordingly, the Bonferroni-adjusted significance levels were 0.004167 (0.05/12) for FABQ-PA and 0.002381 (0.05/21) for FABQ-W.

Targeting was evaluated by comparing the distribution of participant locations with the distribution of item thresholds on the common logit scale. Adequate targeting was considered present when the range of item thresholds closely matched the distribution of person locations, indicating that the subscale appropriately measured the levels of fear-avoidance beliefs represented within the study sample [12,25].

### 2.6. Sample Size Estimation

The required sample size was determined using recommendations specific to studies evaluating structural validity with the Rasch measurement model. Consistent with COSMIN guidance, a sample of at least 100 participants is considered adequate for studies applying the Rasch measurement model to patient-reported outcome measures [32]. This recommendation is supported by previous work demonstrating that a sample of approximately 100 participants provides sufficiently precise estimation of item locations, with calibration errors generally remaining within ±0.5 logits at the 95% confidence level when the scale is reasonably well targeted to the study population [33]. Based on these recommendations, recruitment aimed to include no fewer than 100 participants.

## 3. Results

A total of 113 participants with LBP were included in the Rasch analyses (Table 1). Most participants in the current study were employed (63.7%) and had chronic LBP (68.1%). The mean FABQ-PA score was 15.24, and the mean FABQ-W score was 17.33 (Table 1). Participant demographic and clinical characteristics are presented in Table 1. One participant had a missing response to FABQ item 3; this response was retained as missing, and no imputation was performed.

### 3.1. Rasch Model Selection

The likelihood-ratio test was statistically significant for both the FABQ-PA (χ^2^(8) = 19.81, *p* = 0.01) and FABQ-W (χ^2^(17) = 27.99, *p* = 0.04), supporting the use of the Partial Credit Model, which allows the threshold structure to vary across items.

### 3.2. FABQ-PA

The initial analysis of the FABQ-PA demonstrated satisfactory overall fit to the Rasch model, with a non-significant item–trait interaction (Table 2). The mean item fit residual was 0.62, and the mean person fit residual was −0.37. Despite satisfactory overall model fit, eight participants demonstrated substantial person misfit based on standardized person fit residuals outside ±2.5 and were removed. The excluded participants were slightly older on average than those retained (37.38 vs. 30.54 years) and had lower mean FABQ-PA scores (12.50 vs. 15.45). Among the excluded participants, 62.50% were female and 75.00% had chronic LBP, compared with 51.40% and 67.60%, respectively, among those retained. Following their removal, the final analysis included 105 participants and continued to demonstrate satisfactory overall model fit with a non-significant item–trait interaction (Table 2). The mean item fit residual was 0.57, and the mean person fit residual was −0.19 (Table 2).

All four FABQ-PA items demonstrated satisfactory individual item fit in the final solution, with fit residuals ranging from 0.22 to 0.98 and no statistically significant item-level chi-square statistics (Table 3). Item locations ranged from −0.10 logits for item 4 (“I should not do physical activities which (might) make my pain worse”) to 0.17 logits for item 5 (“I cannot do physical activities which (might) make my pain worse”) (Table 3).

**Figure 1:**
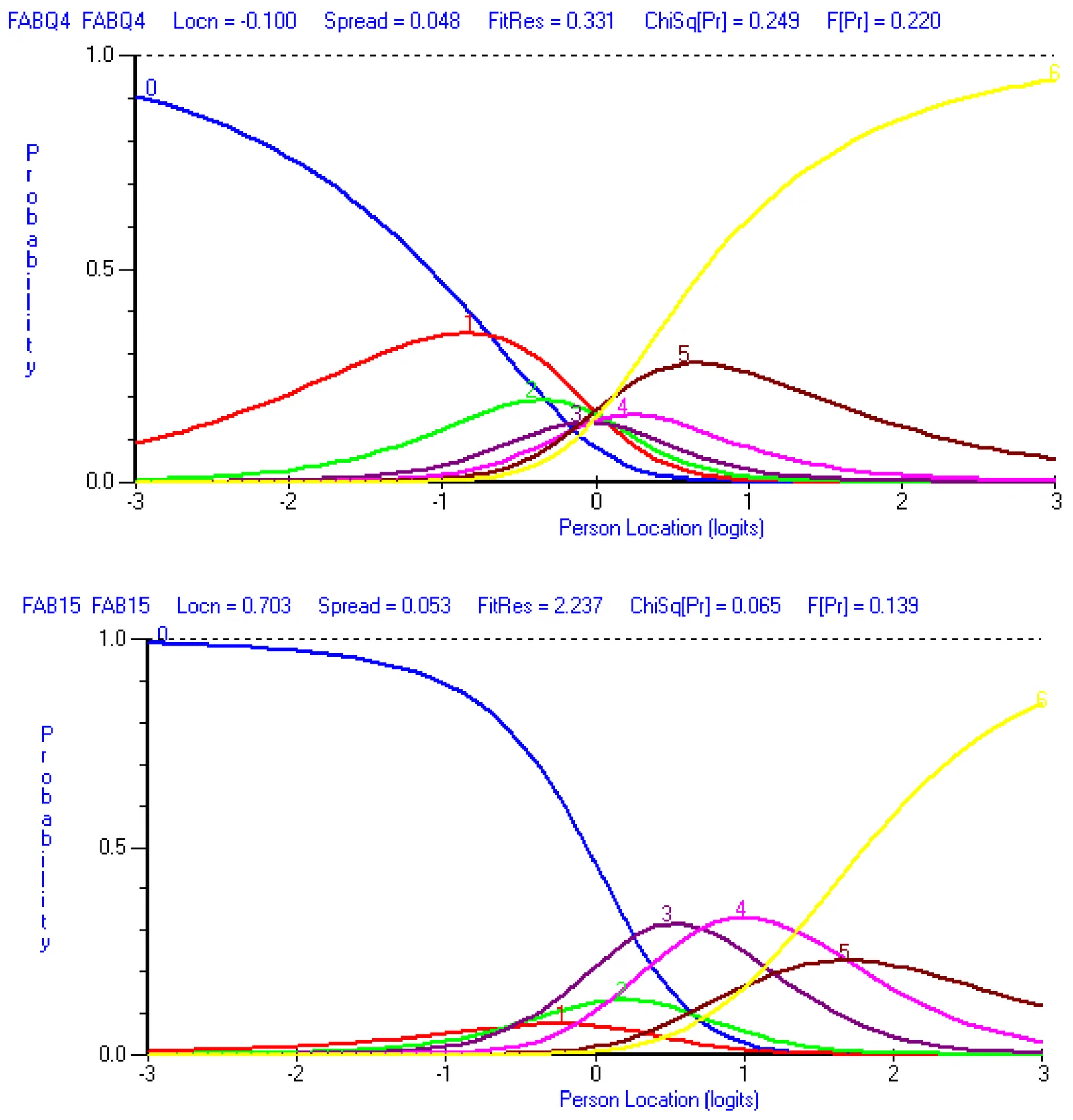
Category probability curves illustrating disordered response-category thresholds under the original seven-category response scale. The (**upper panel**) presents FABQ-PA item 4, and the (**lower panel**) presents FABQ-W item 15. The curves represent the probabilities of endorsing the respective response categories across the latent trait continuum. FABQ-PA, Fear-Avoidance Beliefs Questionnaire–Physical Activity subscale; FABQ-W, Fear-Avoidance Beliefs Questionnaire–Work subscale.

**Figure 2:**
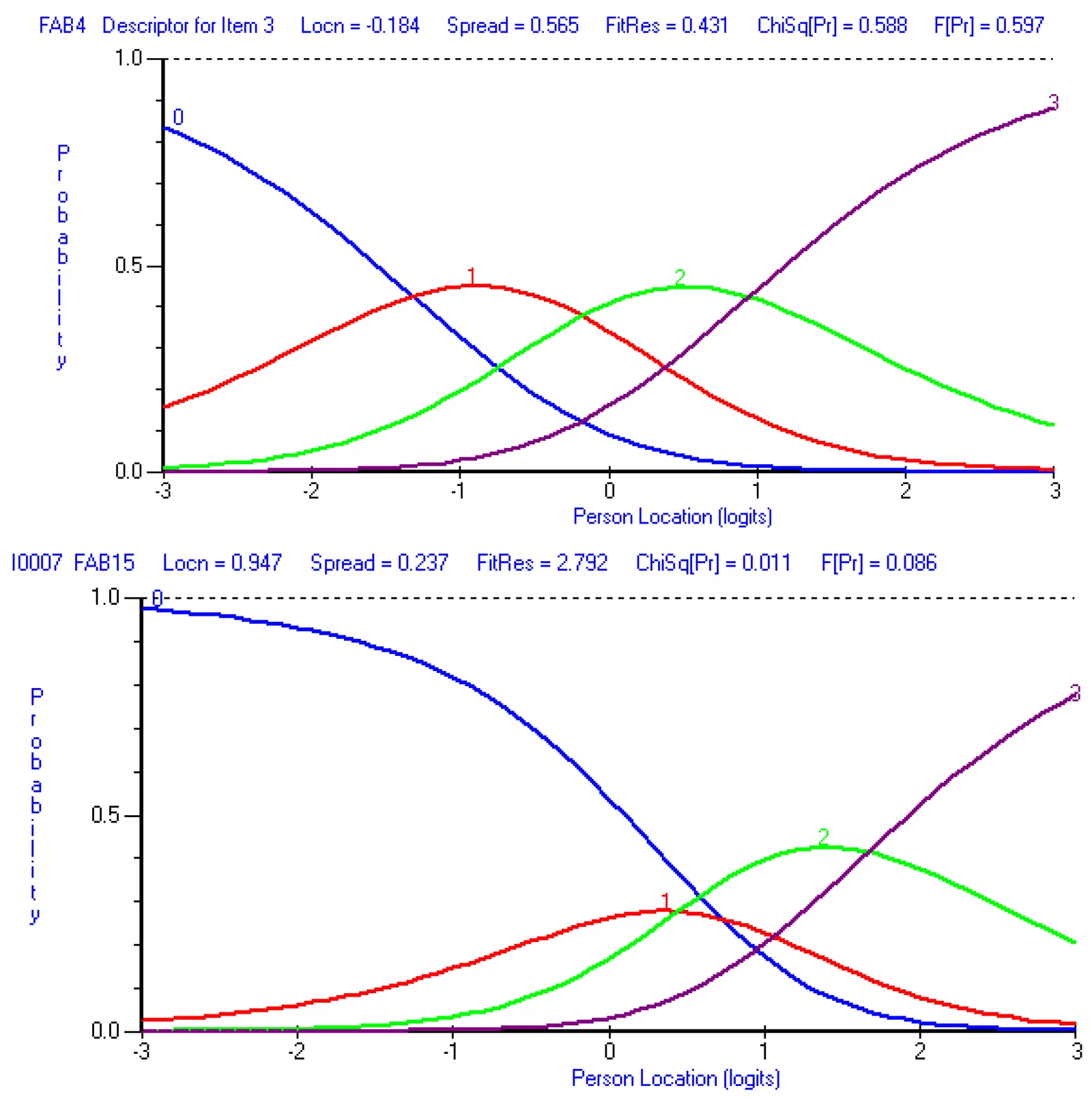
Category probability curves following application of the 011X223 rescoring structure. Under this structure, original category 0 remained scored as 0; categories 1 and 2 were combined and scored as 1; category 3 (“unsure”) was treated as missing (X); categories 4 and 5 were combined and scored as 2; and category 6 was scored as 3. The curves represent the probabilities of endorsing the respective rescored response categories across the latent trait continuum. Although the rescoring improved category functioning (item 3; **upper panel**), disordered thresholds remained for some FABQ items (item 15; **lower panel**).

Despite satisfactory model and individual item fit, examination of the category probability curves revealed disordered thresholds for all four FABQ-PA items, indicating that the original seven response categories did not function in the intended ordinal sequence. Figure 1 presents item 4 as an example of the disordered response category functioning. Given the systematic threshold disordering, a recently proposed four-category rescoring structure (011X223) was subsequently examined [18]. Under this structure, original response category 0 (completely disagree) remained scored as 0, categories 1 and 2 were combined and scored as 1, category 3 (unsure) was treated as missing, categories 4 and 5 were combined and scored as 2, and category 6 (completely agree) was scored as 3. Thus, the rescored structure comprised four scored categories (0–3), with the original ‘unsure’ category excluded from estimation. Although this rescoring improved response category functioning, it did not completely resolve the threshold disordering, with item 5 continuing to demonstrate disordered thresholds after rescoring. Figure 2 provides an example of response category functioning following application of the proposed rescoring structure.

No evidence of local item dependency was identified in the final FABQ-PA solution. The largest residual correlation was observed between items 2 and 3 (r = 0.07), which was below the prespecified criterion for local dependency. No DIF was detected according to sex (male, n = 51; female, n = 54), age (≤28 years, n = 56; >28 years, n = 49), or LBP duration (acute/subacute, n = 34; chronic, n = 71). Given the subgroup sizes; however, the analysis may have had limited ability to detect small DIF effects. The PSI was 0.60 in the final analysis, indicating relatively limited person separation (Table 2). The PSI changed only slightly from 0.62 in the initial analysis to 0.60 after removal of the misfitting participants (Table 2). Assessment of unidimensionality using Smith’s approach supported the unidimensional structure of the FABQ-PA. The positive residual-loading subset comprised items 4 and 5, and the negative residual-loading subset comprised items 2 and 3. None of the *t*-tests comparing person estimates derived from the positively and negatively loading item subsets was statistically significant (Table 2).

**Figure 3:**
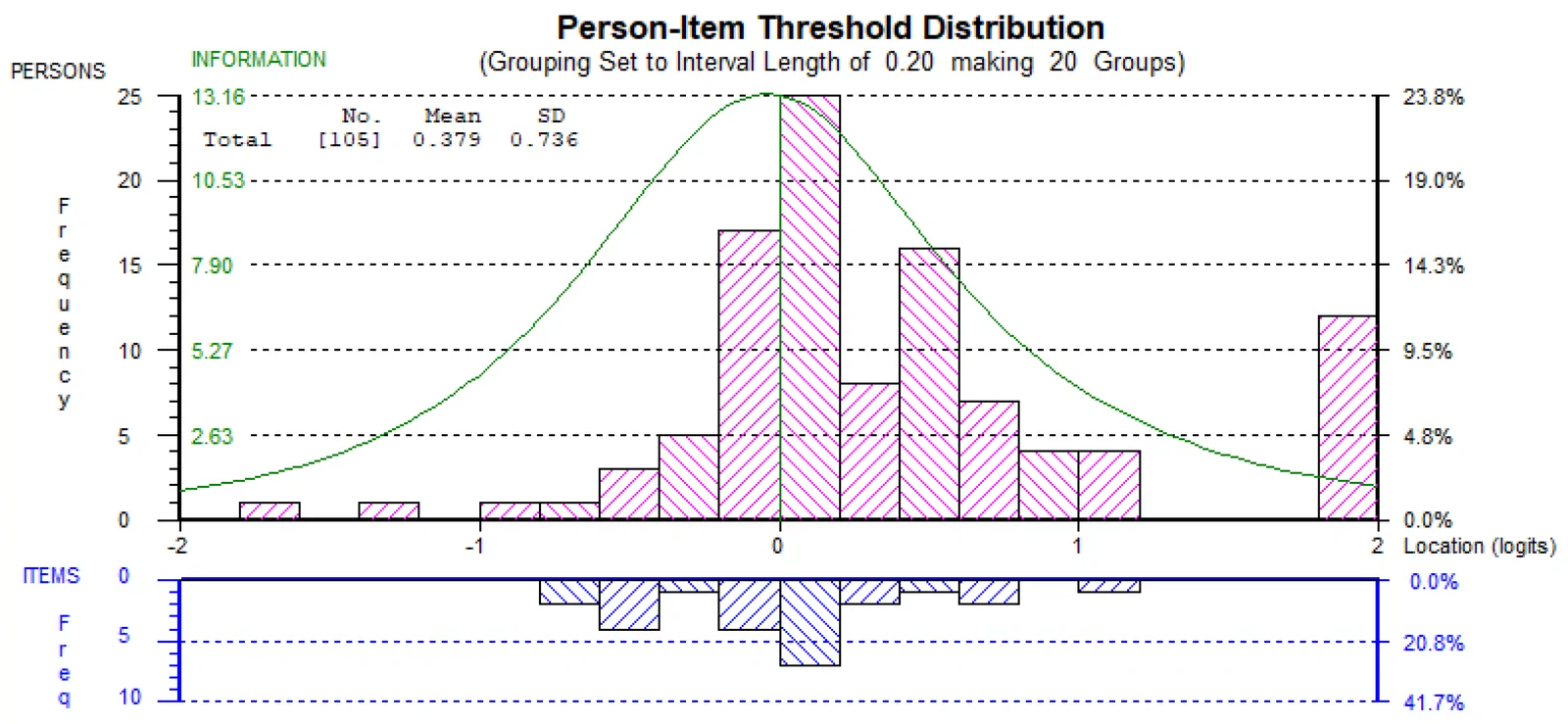
Person–item threshold distribution and subscale information function for the FABQ-PA. In the upper panel, the vertical axis represents frequency, with the distribution above the horizontal axis representing person locations and the distribution below the horizontal axis representing item-threshold locations. Person and item-threshold locations are expressed on the Rasch logit scale along the horizontal axis, with higher values indicating higher levels of physical activity-related fear-avoidance beliefs. The green line presents the subscale information function across the same latent trait continuum, with the vertical axis representing test information. FABQ-PA, Fear-Avoidance Beliefs Questionnaire–Physical Activity subscale.

The person–item threshold distribution indicated generally adequate targeting of the FABQ-PA to the study sample (Figure 3). The mean person location was 0.379 logits (SD = 0.736), reasonably close to the item mean of zero logits, with substantial overlap between the distributions of person locations and item thresholds. Coverage was less optimal at the upper end of the construct, where some participants extended beyond the range covered by most item thresholds. Consistent with this distribution, the subscale information function was greatest around the central region of the latent trait and decreased toward the higher end of the person distribution (Figure 3).

### 3.3. FABQ-W

The initial analysis of the FABQ-W demonstrated a significant item–trait interaction (χ^2^(14) = 30.01, *p* = 0.01), indicating inadequate overall fit to the Rasch model. The mean item fit residual was 0.42, and the mean person fit residual was −0.27. Six participants demonstrated substantial person misfit based on standardized person fit residuals outside ±2.5 and were removed, resulting in a sample of 107 participants. Following their removal, overall model fit improved and the item–trait interaction was no longer statistically significant (χ^2^(14) = 22.21, *p* = 0.07). One additional participant demonstrating substantial person misfit was subsequently identified and removed. The excluded and retained participants were similar in mean age (31.14 vs. 31.02 years) and mean FABQ-W scores (18.57 vs. 17.25). Among the excluded participants, 42.9% were female and 42.9% had chronic LBP, compared with 52.8% and 69.8%, respectively, among those retained. The final analysis included 106 participants and demonstrated satisfactory overall model fit (χ^2^(14) = 22.14, *p* = 0.08). The final mean item fit residual was 0.31, and the mean person fit residual was −0.16 (Table 2).

All seven FABQ-W items demonstrated satisfactory individual item fit in the final solution. Item fit residuals ranged from −0.96 for item 7 to 2.24 for item 15, with all values within the prespecified ±2.5 criterion and no statistically significant item-level chi-square statistics (Table 3). Item locations ranged from −0.43 logits for item 7 to 0.70 logits for item 15, representing the lowest and highest item locations, respectively (Table 3).

As with the FABQ-PA, all seven FABQ-W items demonstrated disordered thresholds under the original seven-category response structure. Figure 1 presents item 15 as an example of the disordered response category functioning. Application of the previously proposed 011X223 rescoring structure improved response category functioning but did not fully resolve the threshold disordering. Four items (6, 7, 11, and 15) continued to demonstrate disordered thresholds following rescoring. Figure 2 provides an example of response category functioning following implementation of the proposed rescoring structure.

No evidence of local item dependency was detected in the final FABQ-W solution. The largest residual correlation was observed between items 6 and 7 (r = 0.06), which was below the prespecified criterion. No DIF was detected according to sex (male, n = 50; female, n = 56), age (≤28 years, n = 55; >28 years, n = 51), or LBP duration (acute/subacute, n = 32; chronic, n = 74). Given the subgroup sizes, however, the analysis may have had limited ability to detect small DIF effects. The PSI was 0.80 in the final analysis, indicating good person separation (Table 2). The PSI remained unchanged at 0.80 following removal of the misfitting participants. Assessment of unidimensionality using Smith’s approach supported the unidimensional structure of the FABQ-W. The positive residual-loading subset comprised items 9, 12, and 15, and the negative residual-loading subset comprised items 6, 7, 10, and 11. Six of the 106 *t*-tests comparing person estimates derived from these subsets were statistically significant (5.66%; 95% CI, 2.62–11.80%). Because the 95% confidence interval included the prespecified 5% criterion, the findings supported unidimensionality (Table 2).

**Figure 4:**
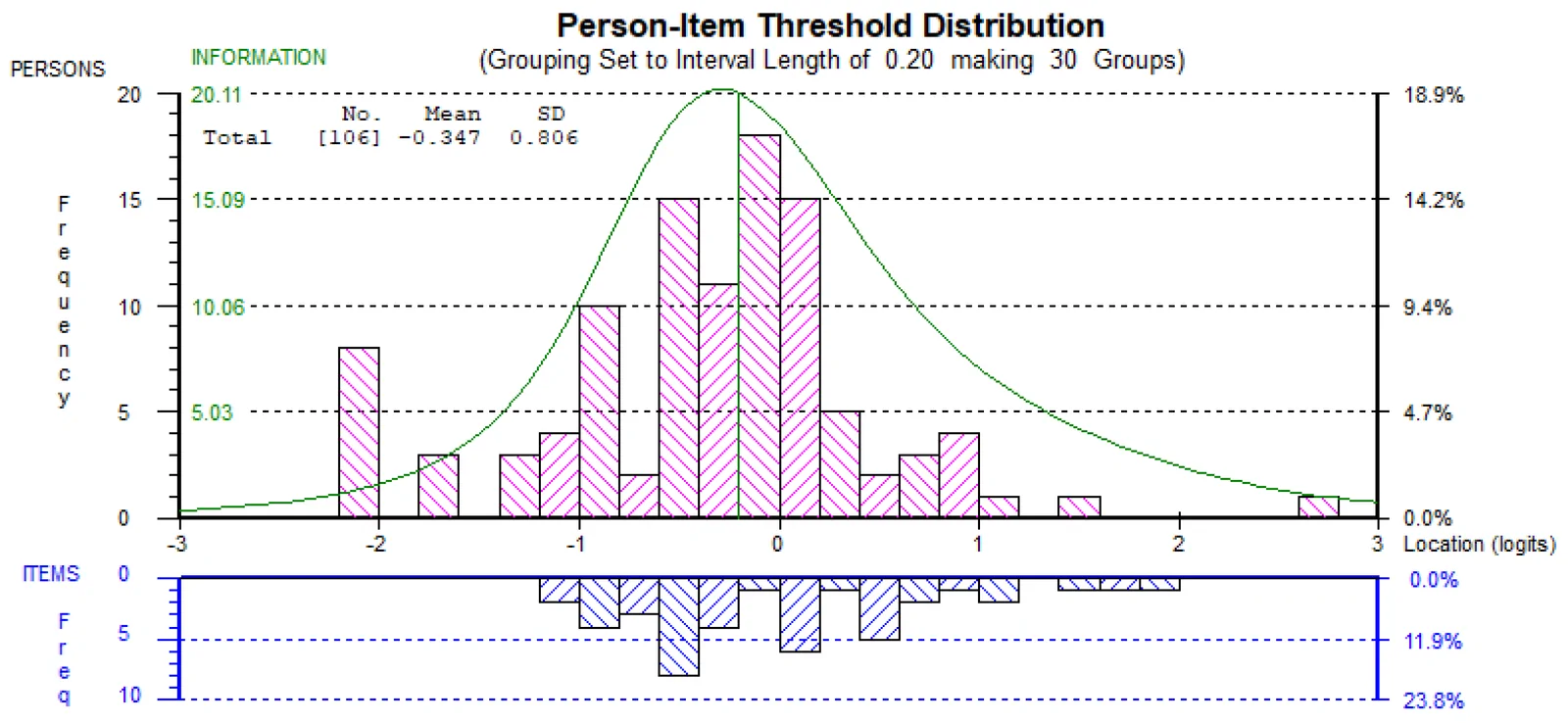
Person–item threshold distribution and subscale information function for the FABQ-W. In the upper panel, the vertical axis represents frequency, with the distribution above the horizontal axis representing person locations and the distribution below the horizontal axis representing item-threshold locations. Person and item-threshold locations are expressed on the Rasch logit scale along the horizontal axis, with higher values indicating higher levels of work-related fear-avoidance beliefs. The green line presents the subscale information function across the same latent trait continuum, with the vertical axis representing test information. FABQ-W, Fear-Avoidance Beliefs Questionnaire–Work subscale.

The person–item threshold distribution also indicated generally adequate targeting of the FABQ-W to the study sample (Figure 4). The mean person location was −0.347 logits (SD = 0.806), reasonably close to the item mean of zero logits, and the item thresholds covered a substantial proportion of the observed person distribution. Coverage was less optimal toward the lower end of the construct, where some participants with lower work-related fear-avoidance beliefs extended beyond the range covered by most item thresholds. The subscale information function was concentrated around the central region of the latent trait, with decreasing information toward the lower and upper extremes of the person distribution.

## 4. Discussion

The present study examined the measurement properties of the Arabic FABQ-PA and FABQ-W in individuals with LBP using the Rasch measurement model. The findings were mixed. Following removal of participants demonstrating substantial person misfit, both subscales showed satisfactory final overall and individual item fit, supported unidimensionality, and showed no evidence of local item dependency or detected DIF according to sex, age, or LBP duration. Targeting was generally adequate, although measurement coverage was less optimal at the higher end of physical activity-related fear-avoidance beliefs and the lower end of work-related fear-avoidance beliefs. However, FABQ-PA demonstrated limited person separation, and all items in both subscales showed disordered thresholds with the original seven-category response scale. Moreover, the previously proposed four-category rescoring structure improved but did not completely resolve threshold disordering. These findings indicate important limitations in the measurement performance of the original FABQ response format that should be considered when interpreting scores.

The present findings add to a relatively small and conflicting body of literature examining the FABQ using Rasch methods. Meroni et al. [16] were the first to apply Rasch analysis to the FABQ in individuals with chronic LBP and reported substantial problems with model fit and response-category functioning. Their analyses did not support satisfactory unidimensional measurement by the FABQ subscales, leading the authors to recommend caution in interpreting their summed scores. Aasdahl et al. [17] subsequently reported similarly unfavorable findings in a considerably larger sample of 722 individuals undergoing occupational rehabilitation. Neither FABQ-PA nor FABQ-W adequately fitted the Rasch model, with three of four FABQ-PA items and three of seven FABQ-W items demonstrating misfit. They additionally identified local dependency and DIF for some items and concluded that neither subscale adequately represented a unidimensional construct.

In contrast, Franchignoni et al. [18] reported more favorable results in 155 adults with chronic LBP. After confirming two dimensions corresponding to FABQ-PA and FABQ-W and analyzing the subscales separately, they found evidence supporting their essential unidimensionality following modification of the response scale and accommodation of local dependency in FABQ-W. Their findings suggested that the fundamental two-subscale structure may be defensible despite problems with the original response format. The present findings are more consistent with this latter interpretation. Both Arabic subscales demonstrated satisfactory final overall and item-level fit and supported unidimensional measurement, suggesting that the four physical activity items and seven work items can each represent a common underlying construct in this population.

An important distinction between the present study and the earlier Rasch investigations concerns individual item performance. All four FABQ-PA items and all seven FABQ-W items demonstrated satisfactory fit in the final Arabic solutions, without requiring item deletion. This differs notably from Aasdahl et al. [17], who reported substantial item misfit in both subscales, and from Meroni et al. [16], whose analyses did not yield satisfactory Rasch solutions. Franchignoni et al. [18] obtained findings closer to those of the present study, although their final solution still contained an overfitting FABQ-PA item and a slightly underfitting FABQ-W item. These differences suggest that the performance of individual FABQ items may potentially be influenced by clinical, linguistic, and cultural characteristics. This reinforces the importance of examining translated patient-reported outcome measures within their intended populations rather than assuming that measurement properties established in one language or population necessarily generalize to another.

The most consistent finding across the present study and the three previous Rasch investigations concerns the FABQ response scale. All four FABQ-PA items and all seven FABQ-W items in the Arabic version demonstrated disordered thresholds, indicating that respondents did not consistently use the seven response categories as progressively ordered levels of the underlying constructs. Meroni et al. [16] similarly reported disordered thresholds, while Aasdahl et al. [17] identified disordered thresholds in all analyzed items and found that only one or two thresholds were distinguishable for each item. They consequently characterized the original seven-point scoring structure as excessively fine-grained for their population. The convergence of this finding across studies conducted in different countries and languages is noteworthy: despite disagreement regarding dimensionality and overall Rasch fit, all four investigations indicate that respondents have difficulty reliably distinguishing among the seven original FABQ response levels.

There are plausible reasons for this finding. The original response scale contains seven numerical categories but only three explicit verbal anchors: 0 = “completely disagree,” 3 = “unsure,” and 6 = “completely agree” [5]. Respondents therefore have to infer the meaning of categories 1, 2, 4, and 5. The Rasch measurement model principles require successive categories to represent ordered and distinguishable levels of the underlying construct; adding response options that respondents cannot reliably discriminate may introduce noise rather than additional measurement information [34]. Research on rating scales more generally has also demonstrated that midpoint categories such as “unsure” may be used for heterogeneous reasons, including genuine neutrality, uncertainty, indecision, or reluctance to express a directional response, rather than representing a precise intermediate location on the construct continuum [35,36].

Franchignoni et al. [18] attempted to address these problems by collapsing categories 1 and 2 and categories 4 and 5 and treating category 3 (“unsure”) as missing, producing the 011X223 structure. The resulting categories corresponded to completely disagree, disagree, agree, and completely agree and demonstrated ordered functioning in their Italian sample. In the present study, applying the same structure improved category functioning but did not completely resolve the threshold disordering; FABQ-PA item 5 and FABQ-W items 6, 7, 11, and 15 continued to demonstrate disordered thresholds. Therefore, the present results strengthen the argument that the original seven-category FABQ response scale requires reconsideration but do not provide sufficient evidence to endorse the particular four-category solution proposed by Franchignoni et al. [18] for the Arabic version. Accordingly, the proposed four-category rescoring should not currently be adopted as an alternative scoring system for the Arabic FABQ. Until an alternative response format is prospectively validated, clinicians and researchers may continue to use the established scoring of the Arabic FABQ, but scores should be interpreted cautiously given the observed dysfunction of the original response categories.

The persistence of threshold disordering after rescoring also suggests that a universally applicable post hoc rescoring solution may not exist. Differences in how respondents interpret agreement categories may arise from linguistic and cultural factors as well as clinical characteristics [19]. Consequently, future studies should prospectively investigate alternative FABQ response formats rather than relying exclusively on statistical rescoring of responses obtained using the original format. A smaller number of fully verbally labeled response categories may be particularly worthy of investigation. Cognitive interviewing could also help determine how Arabic-speaking respondents distinguish between adjacent categories and interpret the “unsure” midpoint before a revised response structure is formally evaluated using Rasch methods [37].

No DIF was detected according to sex, age, or LBP duration in the present sample, suggesting that no substantial subgroup-related item bias was identified for the characteristics examined. However, given the relatively modest subgroup sizes, these findings should be interpreted cautiously because the analyses may have had limited ability to detect small DIF effects. This is an important property because DIF may compromise the comparability of scores across groups even when an instrument demonstrates satisfactory overall fit [38]. The present findings agree closely with Franchignoni et al. [18], who found no significant DIF according to sex, age, or pain duration. Aasdahl et al. [17] similarly reported invariance according to age but detected gender DIF for two items. More broadly, Hart et al. [39], using item–response–theory methods in a large outpatient rehabilitation sample, reported absent or generally negligible DIF across several demographic and clinical characteristics. Collectively, these findings provide preliminary evidence that the FABQ items are relatively stable across the patient characteristics examined, although larger samples would provide greater power to detect smaller DIF effects.

Reliability differed meaningfully between the two subscales. The FABQ-W demonstrated a PSI of 0.80, indicating good separation of individuals according to work-related fear-avoidance beliefs, whereas the FABQ-PA PSI of 0.60 indicates more limited discrimination. A similar pattern was reported by Meroni et al. [16] and Franchignoni et al. [18], with person separation index values of 0.61–0.69 for FABQ-PA and 0.75–0.79 for FABQ-W. The consistently lower reliability of FABQ-PA may partly reflect its brevity: with only four items, relatively little information is available to distinguish among respondents with similar levels of the latent trait. This interpretation is consistent with the general measurement principle that reliability and person separation depend partly on the amount and distribution of information provided by the items [12,25]. The FABQ-PA should therefore be used cautiously when precise differentiation between individual patients is required.

The person–item distributions nevertheless indicated generally satisfactory targeting of both Arabic subscales. The mean person locations were relatively close to the item mean of zero logits, suggesting reasonable correspondence between the levels of fear-avoidance beliefs represented by the items and those observed in the sample. However, coverage was less optimal among individuals with high physical activity-related fear-avoidance beliefs for FABQ-PA and those with low work-related fear-avoidance beliefs for FABQ-W. The information functions similarly indicated greatest precision around the central portions of the latent traits and declining information toward the extremes. These targeting limitations indicate that measurement precision may be reduced for individuals at these ends of the respective constructs. For FABQ-PA, the limited coverage at the higher end may partly reflect the small number of items in the four-item subscale and the resulting limited range of item thresholds available to assess individuals with particularly high physical activity-related fear-avoidance beliefs. For FABQ-W, the reduced coverage at the lower end may reflect the content of the work-related items as well as characteristics of the present sample, which included participants with varying work status. However, the present findings cannot determine whether these targeting limitations are primarily attributable to the item content or the characteristics of the sample. Franchignoni et al. [18] reported good targeting for FABQ-PA but poorer FABQ-W targeting, with a mean person location of −1.51 logits, indicating that their FABQ-W items were comparatively better targeted to individuals with higher levels of work-related fear-avoidance beliefs. The present FABQ-W targeting therefore appears more favorable overall, although both studies suggest that measurement at the lower end of work-related fear-avoidance beliefs could potentially be improved.

The discrepancies among the four Rasch studies should be interpreted in light of differences in populations and analytical procedures. Both Meroni et al. [16] and Franchignoni et al. [18] examined Italian adults with chronic LBP, whereas the present sample included acute, subacute, and chronic LBP, although chronic presentations predominated. Aasdahl et al. [17] studied a much broader occupational rehabilitation population that included musculoskeletal, unspecified, and common mental health conditions, and part of their sample completed a modified FABQ in which “complaints” replaced “pain” and “body” replaced “back.” The studies also differed in model selection, fit criteria, handling of local dependency, and approaches to category restructuring. Notably, Franchignoni et al. [18] selected the rating scale model because their model comparison did not favor the partial credit model, whereas the likelihood-ratio tests in the present study supported use of the partial credit model. Such methodological and sample differences may partly explain why studies using the same questionnaire have reached different conclusions regarding Rasch model fit.

In practical terms, clinicians and researchers may continue to use the established Arabic FABQ scoring system but should interpret scores cautiously because of the disordered response-category thresholds, while FABQ-PA should not be relied upon when precise differentiation between individuals with similar levels of physical activity-related fear-avoidance beliefs is required.

Several strengths should be acknowledged. To our knowledge, this is the first study to examine the Arabic FABQ using the Rasch measurement model. A comprehensive set of measurement properties was investigated, including model selection, overall and individual item fit, response-category functioning, local independence, DIF, reliability, unidimensionality, and targeting. Furthermore, analyzing FABQ-PA and FABQ-W separately was consistent with the intended scoring of the original instrument [5], previous factor-analytic evidence, and the recent CFA of the Arabic version [11]. The present sample substantially overlaps with that used in our previous CFA of the Arabic FABQ [11]; however, the two studies address distinct but complementary measurement questions using different analytical frameworks. Several limitations should also be considered. First, participants were recruited from outpatient physical therapy settings in the central region of Saudi Arabia, potentially limiting generalizability to Arabic-speaking populations in other regions and healthcare settings. Given that linguistic and cultural factors may influence the interpretation of response categories, future studies should examine the measurement properties and response-category functioning of the Arabic FABQ in different Arabic-speaking countries, regions, and clinical populations. Second, although the sample was adequate for the primary Rasch analyses, subgroup sizes were smaller for DIF testing, which may have limited the ability to detect small DIF effects. Therefore, the absence of detected DIF should be interpreted cautiously and should not be considered definitive evidence of measurement invariance across the examined subgroups. Third, participants demonstrating substantial person misfit were removed in obtaining the final Rasch solutions. Although examination of person misfit is an established component of Rasch analysis [12,25], the final model-fit statistics apply to the retained samples and should be interpreted accordingly. This consideration is particularly relevant to FABQ-W, for which the initial analysis showed inadequate overall model fit and satisfactory fit was obtained after removal of misfitting participants. In contrast, FABQ-PA demonstrated satisfactory overall fit before person exclusions, and person removal had little influence on the PSI of either subscale. Replication in independent samples would help determine the stability of these findings without reliance on person exclusions. In addition, not all participants were currently employed. Although all participants completed the original FABQ-W as written, current work participation may influence the relevance or interpretation of work-related items. The potential influence of work status on FABQ-W response-category functioning and targeting should therefore be examined in future studies. Finally, the study was cross-sectional and consequently does not establish longitudinal invariance or responsiveness of the Rasch-derived measures.

## 5. Conclusions

The Arabic FABQ-PA and FABQ-W demonstrated satisfactory final Rasch model and item fit, supported unidimensionality, showed no meaningful local item dependency or detected DIF, and were generally well targeted to the study sample. However, important measurement limitations were identified. FABQ-PA demonstrated limited person separation, and all items in both subscales showed disordered thresholds with the original seven-category response format. The previously proposed four-category rescoring structure improved but did not fully resolve threshold disordering and therefore cannot currently be recommended as a validated alternative for the Arabic FABQ. These findings do not preclude continued use of the FABQ-PA and FABQ-W as separate subscales; however, scores should be interpreted cautiously, particularly given the response-category dysfunction and limited measurement precision of FABQ-PA. Further investigation and refinement of the FABQ response format are warranted.

## Figures and Tables

**Table 1:** Demographic and Clinical Characteristics of the Study Participants.

Variable	Mean ± SD or N (%)
Sex	
Male	54 (47.79)
Female	59 (52.21)
Age (years)	31.03 ± 12.49
Age group	
≤28 years	57 (50.44)
>28 years	56 (49.56)
Height (m)	1.67 ± 0.10
Mass (kg)	71.54 ± 12.14
Body mass index (kg/m^2^)	25.96 ± 5.61
LBP duration	
Acute (<1 month)	17 (15.04)
Subacute (1–3 months)	19 (16.81)
Chronic (>3 months)	77 (68.14)
Diagnosis	
Disk-related disorders	32 (28.32)
Non-specific/mechanical LBP	48 (42.48)
Spinal degenerative/structural disorders	31 (27.43)
Sacroiliac disorders	2 (1.77)
Work Status	
Employed	72 (63.72)
Housework	20 (17.70)
Unemployed	18 (15.93)
Retired	3 (2.65)
FABQ-PA (0–24)	15.24 ± 5.51
FABQ-W (0–42)	17.33 ± 10.52
NPRS (0–10)	5.50 ± 2.04
ODI (0–100)	25.27 ± 15.38

LBP = Low back pain; FABQ-W = Fear-avoidance beliefs questionnaire-work subscale; FABQ-PA = Fear-avoidance beliefs questionnaire-physical activity subscale; NPRS = Numeric Pain Rating Scale; ODI = Oswestry Disability Index.

**Table 2:** Summary of Rasch Model Fit, Reliability, and Unidimensionality Analyses for the FABQ-PA and FABQ-W Subscales.

Run	Analysis	N	Item Fit Residual		Person Fit Residual		Item–Trait Interaction		PSI	Unidimensionality *t*-Tests	
			Mean	SD	Mean	SD	χ^2^ (df)	*p*		Significant Tests n/N (%)	95% Binomial CI
Fear-avoidance beliefs questionnaire-physical activity subscale (FABQ-PA)											
1	Initial analysis	113	0.62	0.39	−0.37	1.28	6.41 (8)	0.60	0.62	2/113 (1.77%)	0.49–6.22%
2	Delete 8 misfit persons	105	0.57	0.35	−0.19	0.93	6.24 (8)	0.62	0.60	0/105 (0.00%)	0.00–3.53%
Fear-avoidance beliefs questionnaire-work subscale (FABQ-W)											
1	Initial analysis	113	0.42	1.59	−0.27	1.23	30.01 (14)	0.01	0.80	8/113 (7.08%)	3.63–13.35%
2	Delete 6 misfit persons	107	0.33	1.21	−0.17	0.98	22.21 (14)	0.07	0.80	6/107 (5.61%)	2.60–11.70%
3	Delete 1 misfit person	106	0.31	1.20	−0.16	0.95	22.14 (14)	0.08	0.80	6/106 (5.66%)	2.62–11.80%

SD = standard deviation; χ^2^ = chi-square; df = degrees of freedom; PSI = Person Separation Index; CI = confidence interval; FABQ-PA = Fear-Avoidance Beliefs Questionnaire–Physical Activity subscale; FABQ-W = Fear-Avoidance Beliefs Questionnaire–Work subscale.

**Table 3:** Individual Item Locations and Fit Statistics for the FABQ-PA and FABQ-W Subscales.

Item	Location	SE	Fit Residual	χ^2^	*p*
Fear-avoidance beliefs questionnaire-physical activity subscale (FABQ-PA)					
2. Physical activity makes my pain worse	−0.03	0.07	0.72	0.89	0.64
3. Physical activity might harm my back	−0.03	0.07	0.22	2.26	0.32
4. I should not do physical activities which (might) make my pain worse	−0.10	0.06	0.33	2.78	0.25
5. I cannot do physical activities which (might) make my pain worse	0.17	0.06	0.98	0.31	0.86
Fear-avoidance beliefs questionnaire-work subscale (FABQ-W)					
6. My pain was caused by my work or by an accident at work	−0.15	0.06	1.11	0.70	0.71
7. My work aggravated my pain	−0.43	0.07	−0.96	4.04	0.13
9. My work is too heavy for me	0.36	0.07	−0.37	2.75	0.25
10. My work makes or would make my pain worse	−0.28	0.07	−0.34	4.31	0.12
11. My work might harm my back	−0.26	0.07	−0.68	2.01	0.37
12. I should not do my normal work with my present pain	0.05	0.07	1.20	2.84	0.24
15. I do not think that I will be back to my normal work within 3 months	0.70	0.08	2.24	5.48	0.06

SE = standard error; χ^2^ = chi-square; FABQ-W = Fear-Avoidance Beliefs Questionnaire–Work subscale; FABQ-PA = Fear-Avoidance Beliefs Questionnaire–Physical Activity subscale. Item locations are expressed in logits, with higher positive values representing higher levels of the corresponding fear-avoidance construct. Item-level *p*-values are unadjusted values; statistical significance was evaluated using Bonferroni-adjusted significance levels of 0.0125 (0.05/4 items) for FABQ-PA and 0.0071 (0.05/7 items) for FABQ-W.

## Data Availability

The data presented in this study are not publicly available due to privacy and ethical restrictions but are available from the corresponding author upon reasonable request.
